# Dr. Ratier on the Hospital Practice of Paris

**Published:** 1825-07-01

**Authors:** 


*Formulaire Pratique des Hôpitaux Civiles de Paris, &c. &c. i. e. A Practical Formulary of the Civil Hospitals in Paris; or a Collection of the Medical Prescriptions used by the Physicians and Surgeons of these establishments; with Notes on the Doses and the Modes of Administration of Particular Remedies; and General Observations on each Hospital, on the Kind of Diseases which it is destined to receive, and on the Doctrines of the Practitioners, under whose charge it is placed.* By M. F. Ratier, M.D. &c. &c. 12mo. pp. cvi. 376. Paris, 1823.

Dr. Ratier professes, in this unambitious volume, to describe the state of medical practice in the hospitals of the French metropolis; and, in executing this delicate task, has with great propriety restricted himself to the office of an historian instead of that of a censor or a panegyrist. This plan, of course, imposes on him the necessity of abstaining from the expression of his own sentiments, relative to the importance of those medicines, of which he does not approve, either the composition or the application; and, by the same rule, in like manner, he holds himself absolved from all responsibility, except what regards the accuracy of facts. Having, moreover, been favoured by communications, in writing, from several of the chief officers of these hospitals, he appears to have carefully distinguished these, and given them to his readers without alteration of any kind. In other instances, he considers the practice of each hospital as general, except when a prescription common to all the physicians of that establishment, receives on the part of some individual a particular application. There is much candour as well as good sense in this plan of the doctor's; and we can perceive in it a decided testimony of the advantages which medicine shall at all times derive from the philosophy of facts, in preference to the fictions of speculation.

In most of the Parisian hospitals, the physicians and surgeons employ conventional and abridged terms in their prescriptions, as *eau benite, julep bechique*, for instance; and, in this way, occasion much embarrasment to persons attending their visits, whether for observation or improvement. Our considerate author has essayed to obviate these inconveniences, by collecting into his work the different formulæ designated by such enigmatical expressions; and, at the same time, to explain their doses and mode of administration.

Dr. Ratier asks, in his introduction, if we ought to ascribe to the "new physiological doctrine" (that of M. Broussais, we presume), the great simplicity which is now obtaining in the treatment of diseases; or, if the *medical constitution* itself has undergone a change, as many physicians, especially M. Jadelot, are disposed to assert; or, if gastro-intestinal inflammations have become more frequent in proportion to the decrease of *adynamic* and *ataxic* fevers? It is quite time, he adds, (and the registers of medical experience attest its truth,) that physicians have now much restricted the exhibition of emetics, purgatives, and tonics of every kind, and that much importance is attached, in these days, to inflammations of the digestive canal. Such changes may possibly have occurred in nature, as would seem to be implied in Dr. R's interrogatories; but, it is no less painful than humiliating to find that, in many instances, a mere change of words and names, without the least alteration of circumstances, constitutes the amount of originality in many of the new medical doctrines. In regard to the restriction of evacuants, we suspect Dr. R. has allowed himself to be satisfied with a partial or imperfect view of their importance: on our side of the channel, at least, such means possess a manifest superiority over ptisans of all kinds, or the orange-flower-water, or leeches to unseemly parts, or even to the heroic practice of moxibustion. With these introductory remarks, we pass to the subjects of the text before us: they are interesting in all respects; and, on this account, we shall devote a due degree of attention to the consideration of their nature and usefulness.

We propose adopting Dr. Ratier's arrangement in our analytical view of the plan and execution of his work: it is divided into five sections, containing,—general considerations on the ten hospitals of Paris,—the "formulary" strictly so called,—an account of particular remedies,—a comprehensive and rather elegant "nosology,"—and a copious, and consequently very convenient index, which it were well if many medical writers would choose to imitate.—For the purpose of making our readers acquainted with the economy of French hospital practice, we shall make each of the first three of these divisions the topic of particular examination.

I. General Considerations.—Thanks, says Dr. R. to the cares of a wise and beneficent government, the hospitals of Paris, destined originally to offer a refuge to misfortune and suffering, are become "des foyers" of medical instruction as pure as it is substantial. In them all, clinical schools are established under the superintendance of the most eminent physicians, whose lectures are eagerly frequented by the students. Every practitioner, we are told, availing himself of his opportunities, turns his attention exclusively to the elucidation of a particular subject by numerous researches and ingenious experiments, and thus contributes much to the advancement of the science of healing. Pathological anatomy, cultivated also with great zeal and address, reveals to them the seats and causes of diseases, and indicates new methods of treatment, whether curative or palliative. Every year, moreover, a medical "Recueil" is published, and contains practical observations made in the hospitals; and, by honourable rewards, encouragement is given to such students as have distinguished themselves by their exertions and their assiduity. By such means, our author concludes, these hospitals have proved the nursery of many practitioners that now enjoy a high reputation as promoters of the medical philosophy.—Let us now take each of these sanctuaries of sorrow and of science under notice, according to the order assigned them in Dr. Ratier's arrangement.

*Hôtel-Dieu.*—This is the most ancient and extensive of the Parisian hospitals, as well as the most important, on account of the clinical instruction of which it is the centre: it is set apart exclusively for the reception of acute diseases. The surgical department in it is entrusted to the charge of Messrs. Marjolin and Dupuytren; but the latter performs the whole actual duties: he visits the patients twice every day, holds gratuitous consultations, and performs all the operations in surgery. The following sketch communicated by Professor Dupuytren himself to Dr. Ratier, will afford some idea of the opinion and practice of this indefatigable surgeon.

"Removal of houses adjoining the Hôtel Dieu; free admission of pure air around and within the edifice; suppression of unsalutary wards; reduction of the number of beds; extending the windows down to the floors; transferring the insane, the old and the infirm, persons having ulcerated sores, cutaneous or other contagious diseases, and pregnant females, to establishments appropriated to their reception; and abundance and variety of every thing requisite for personal apparel and bed-clothes, for surgical dressings, for sustenance and for medicine, with the great degree of order and regularity observed in every department of the institution, have contributed to render the Hôtel Dieu one of the most healthy hospitals in the capital. Here, the senses of sight and smell are not, as heretofore, disagreeably affected: adynamic fevers, produced by air loaded with putrid miasms, no longer occur in such numbers: hospital gangrene also supervenes so unfrequently that the Professor has often been obliged to conclude his course of clinical surgery without being able to shew a single case to the students. Trepanning generally succeeds, except in such instances as analogous causes would affect other important operations: on the contrary, and probably from the effect of precautions taken to promote the admission and purification of the air, inflammatory affections are rather prevalent: in fact, if putrid and malignant fevers, gangrenes and mortifications, have nearly disappeared, thoracic, pulmonary, and peritoneal inflammations abound, and are the causes of death to many persons who expire in the surgical wards. All the bodies of individuals who have died in these wards during the last six years, have without exception been opened, and thus afforded a convincing proof of the doctrine, first stated by Dessault, that the greater number of patients who die under treatment for surgical diseases, perish from internal inflammations, *which very often occur to the amount of two, three or even four in the same person.* "Observation of this fact," says the reporter, "has not been lost in the treatment of surgical diseases: in almost all cases, diluents and refrigerants take place of tonics: leechings and venesections are employed instead of cordials and stimulants: bark is very sparingly exhibited." Fractures are almost always treated by position: some of them, as those of the neck of the thigh-bone and neck of the arm, by this means alone; others by position aided by dressings destined chiefly to prevent motion of the limb. Continued extension of the member is never employed. The *frightful* apparatus for reducing fractures and luxations has been banished for ever, and its place supplied by gentle methods. Herniæ are operated upon, so soon as the patient enters the hospital: cataracts are cured by depression and in bed, which prevents accidents from the patient's being removed after the operation. The number of deaths has lessened: it is 1 in 18, 19, or 20, in common years; the operation of lithotomy succeeds in $\frac{5}{6}$; that for hernia, in $\frac{3}{5}$; that for cataract, in $\frac{7}{8}$; and that for fistula lachrymalis, by the introduction of a canula of gold or platina, in $\frac{19}{20}$ of the cases.

Besides the surgical, there is an extensive medical department in the Hôtel Dieu, conducted by seven physicians, in successive courses of three months each. Among these, M. Recamier is much praised by our author, as a zealous, enterprising, sagacious practitioner, guided by observation in preference to all exclusive views: his attention is particularly directed to researches in the materia medica and pathological anatomy. De Montaign, Petit, Borie, and Geoffrey, are pure Brunonians; and their treatment of disease, of course, is tonic and stimulating. On the other hand, M. Husson adopts the principles of the chief (M. Broussais) of "la medecine physiologique," and even exceeds his master in his opinions. Universally almost, he prescribes low diet, emollient drinks, local and general bleedings, baths, and relaxing applications: he holds in utter proscription nearly every other therapeutical remedy, and his great success for several years has, we are told, confirmed the suitableness of his practice. Asselin again, is remarkable for "la sage expectation" he exercises in the management of his patients: persuaded that Nature is often able to accomplish a cure when undisturbed in her course, he makes it his chief concern to prevent every kind of influence calculated to determine or maintain a morbid state, by appropriate regimen, and especially by the greatest reserve in the employment of "des moyens perturbateurs."

*Hôpital de la Pitié.*—This is, in some sort, subordinate to the Hôtel Dieu and the hospital for venereal patients: convalescents and persons having chronic complaints are sent to it, till the place of their ultimate reception be fixed. One division of it is reserved for "filles publiques" affected with the syphilitic disorder: these are sent thither by the police, and treated by the physicians and surgeons of the last-mentioned establishment. Professor Beclard is surgeon-in-chief to the rest of the house; Doctors Serres and Bally are the physicians: the former is well known for his valuable researches in general and pathological anatomy.

*Hôpital de la Charité.*—This, having been considerably enlarged and supplied with the means of administering baths and fumigations of all kinds, is regarded as the second in importance of the metropolitan hospitals. Professors Boyer and Roux superintend the surgical duties in it, and, at the same time, read lectures in clinical surgery: Fouquier, Lerminier, and Chomel, are the physicians; the latter assists also in M. Fouquier's wards, and at the gratuitous consultations. Professor Fouquier himself gives clinical lectures in "l'hospice de la clinique interne;" but observations on the cases in the wards of La Charité are likewise comprehended in his course, which is attended by a great number of students.

In M. Ratier's estimation, this professor is a great man and a true medical philosopher. M. F. in his practice, employs a prudent caution ("temporisation") by leaving something to be done by the conservative efforts of Nature, and something also by those prompt and energetic "determinations" which change or modify a vicious tendency, and rescue the patient from certain death. He is an attentive and scrupulous observer; frank and discriminating; and distinguished for his accuracy in diagnosis, as well as the general certainty of his prognostic judgments: he is deliberate and minute in the investigation of symptoms; and, at all times, entertains a lively sense of the danger of compromising the dignity of the science by rashness. His system of treatment is simple and rational; and, though he occasionally ventures on the practice of an experiment, he does this with a prudence and reserve which exalt the physician's honour without lessening his great responsibility. Absolutely a stranger to the spirit of system, and treading the path defined by observation and experience, he has for a long time inculcated, in his theoretical and practical lectures, the frequency of inflammatory affections, and insisted on the urgency of subduing them by an antiphlogistic treatment, even when they have passed into a chronic state. It is also a doctrine of his, that the essential fevers of the ancients were often a symptom merely of an undetected inflammation: nevertheless, he admits the existence of essential fevers, *i. e.* fevers in which the state of excitement is general and never shews itself so predominant in one part as to authorise its being considered the cause whereby the febrile phenomena are determined. M. Fouquier, moreover, has long set himself against the abuse of stimulant and tonic applications in the treatment of acute diseases: he cannot, besides, bring his mind to believe that the gangrenous inflammation of the bowels and the skin, which manifests itself in carbuncles and adynamic fever, can have the same nature and require the same curative means as the true inflammation of the same parts, which constitutes erysipelas and dysentery.

Professor Fouquier follows a particular method in many diseases: in the colica pictonum (*C. Rachialgia* of Dr. Good) he deviates from the common practice, and makes such rational modifications in the treatment as long experience has demonstrated to be most efficacious. M. Ratier has made a great oversight in neglecting to disclose these important modifications "qu'exigeait une therapeutique rationelle:" to some minds there may be a sublimity in mystery; but we are to hope that M. Fouquier is of a purer mould. He considers acute rheumatism as an actual inflammation, and treats it as such; but prefers the use of leeches and cataplasms, assisted by mild diaphoretic draughts and tepid baths, to venesections, which give rise to tedious convalescence. This method has often succeeded, in his hands, in cases of chronic rheumatism of the joints, even when they had been partially anchylosed. Generally, in neuralgic affections, he first employs a few bleedings, sometimes venous, sometimes capillary, and then prescribes vesicatories to be applied, not on the course of the diseased nerve, but on the opposite surface of the affected member. The number of nervous disorders, so considerable with those who make superficial observations, is greatly reduced by M. F. who tries as much as possible to connect each series of symptoms with the lesion of some organ: notwithstanding this reduction, however, the affections which he is obliged to denominate *nervous* are still very numerous. He admits the existence of diseases purely nervous, *i. e.* in which our means of investigation have not hitherto discovered any material lesion whereunto they ought to be referred. He professes this opinion relatively to asthma, which M. Rostan regards as depending on aneurism of the heart. M. F. has made experiments with the extracts of virulent plants, as hyoscyamus, belladonna, lactuca virosa, with distilled laurel-water, and the prussic acid, in the treatment of epilepsy, hysteria, hypochondriasis, and other nervous maladies; but with what results, M. Ratier has not chosen to communicate.

Dropsy has been the object of M. Fouquier's particular study; and, relatively to this subject, he has instituted many researches on the action of diuretic medicine, which he has exhibited in much higher doses than most practitioners. Since the experiments of Dr. Segalas, he has prescribed *urea*, and found that it exercises a very powerful influence on the organs which perform the urinary secretion. Dr. F. entertains no particular views concerning the venereal disease; only he thinks, with M. Cullerier, that the "liquor of Van Swieten"* is less exceptionable than it has been represented, and that it is quite incapable of *producing* pulmonary consumption, although it may *hasten its development in persons predisposed to this malady*: its bad effects are referrible to its mal-administration in such cases, and in those of chronic gastro-intestinal inflammation.

An original predisposition to tuberculous or cancerous affections has been the subject of much controversy: M. Fouquier admits that, in most instances, such a predisposition really does exist, although it may be possible that it shall remain undetected, and that the diseases it ordinarily determines may accidentally supervene. In aneurism of the heart and large arteries, his treatment consists of local and general bleedings: most frequently he has recourse to the latter, for the purpose of unloading the vascular system. He repeats them according to the facility with which the blood is regenerated, and continues them to the very last stage of the disease: he has often seen venesection revive a patient threatened with instant suffocation. Such evacuations, far from being favourable to the production of symptomatic anasarca, seem, on the contrary, to promote the resorption of extravasated lymph. When this resolution is slow of being attained and distension of the skin is considerable, he makes punctures with a narrow-pointed lancet so deep as to reach the layers of cellular texture, and thus effects a rapid decrease of the swelling. Long experience has convinced him that there is no reason to apprehend the supervention of gangrene; but it is indispensable to the full success of this operation, that the true skin be perfectly penetrated, instead of merely pricking the epidermis with the point of a lancet. To these means are added a light diet and some diuretics to arrest the progress of infiltration: in fine, several compositions of squill and foxglove are exhibited, under the idea of their exercising diuretic and specific influences, by acting on the sensibility of the heart and diminishing the energy of its contractions.

Dr. Lerminier's opinions on some important points of practice have been communicated by himself to our author, in a general way. He treats continued fever by simple diluents,

---

* ℞. Mur. hydrarg. gr. xvj. alcohol. ℥ss. aq. distil. ℔ j. ℳ et solve. The dose is half an ounce in milk, or a solution of gum arabic, morning and evening; but the mixture of the medicine with the vehicle should not be made till the moment of its being taken, for the purpose of preventing decomposition.

so long as no particular indication requires being fulfilled. If an assemblage of inflammatory symptoms, a plethoric state exists, he prescribes a general bleeding: if sanguineous congestions threaten to accumulate in any particular part, he checks them by an application of leeches. It is the different kinds of congestion that chiefly engage his attention during the progress of fevers: he often places, with advantage, leeches on the neck or behind the ears, even at an advanced period of bad fevers and when there is great prostration of strength. On all occasions of profuse diarrhœa, he moderates or suspends it by applying leeches to the anus: often, on the employment of this means, the general oppression disappears and the forces rally. When real debility exists, however, he has recourse to tonic, combined with revulsive remedies: the preparation usually employed by him is the watery infusion of bark. He exhibits emetics for the removal of bilious symptoms: he regards such medicines as particularly serviceable in shortening the course of the disease. Sulphate of quinine, in his hands, readily cures intermittents; but, in some cases, he has found it producing nervous agitations. He treats acute rheumatism with general bleeding, which is very often repeated; until, indeed, the cupped crust of the blood is no longer formed, or, at least, has become less distinct. Leeches, in rheumatism, subdue pain in the part on which they are applied; but, according to M. Lerminier's experience, it soon re-appears in another place: this does not happen, he thinks, after abstracting blood from a vein. Frequently, he promotes the resolution of certain pneumonic complaints which shew a tendency to pass into a chronic state, by substituting for simple emollients, a course of mild tonics, especially the decoction of the rattlesnake-root and antimoniated lohocks. In metallic colics (col. pict.) he follows, to its full extent, the ancient treatment of the "Pères de la Charité," (this is described in the last section) and has found it successful in cases accompanied by a manifest febrile movement.

M. Chomel's practice approximates very nearly that of M. Fouquier; only he is a more zealous partisan of the doctrine of fevers and of the use of tonics and stimulants. This physician has established facts and even performed cures, by means of his method of treatment, on which, says M. Ratier, enlightened and impartial practitioners alone have a right to pronounce an opinion.

*Hôpital Saint Louis.* Persons having cutaneous diseases, and those suffering from scrofulous, scorbutic, and cancerous affections, are received into this institution, where, also, public consultations are held and advice given to out-patients, who obtain tickets authorising them, each day, to take baths and fumigations adapted to the nature of their diseases. Its surgical wards are under the management of Professor Richerand and M. Jules Cloquet: to the charge of Professor Alibert, M. Biett, Manry, and Lugol, the medical department is entrusted.

M. Alibert gives a clinical course, every year, on the diseases of the skin; and, on proper occasions, exhibits the most remarkable cases of such affections to the class. Entrusted successively with the charge of the wards appointed for the reception of persons having the itch, the physicians of this hospital have made numerous and diversified experiments with the view of ascertaining the true nature of this disease, and on the various modes of treatment to which it has been subjected. Dr. Manry has undertaken an investigation for discovering what remedy ought to be preferred, with regard to its expense, its effects on the patient's dress, its odour, and its local and general influences. He has made experiments with twenty-two preparations, and each of these on an equal number of individuals; and, by reckoning the length of the treatment in all the cases, he has assigned the mean period as that of each method. From an account of these proceedings, drawn up "with great exactness and talent," by M. Mélier, a house-pupil, our author has extracted divers formulæ and notes which we find scattered through a subsequent section of his work. Now, with reference to this mode of book-making, we must say, that there is little courtesy in it, and still less philosophy: simple prescriptions, a bare exhibition of the composition of drugs, when compared with an outline of scientific pathology, is, in our estimation, a thing exceedingly insignificant.

M. Ratier also professes having derived advantage from a work on itch,* by M. Mouronval, whose experiments were much more extensive, both in the number of patients and the diversity of remedies employed. Instead, however, of instructing us with a sketch of these interesting reports our author contents himself with telling us, "*en passant*," that M. Mouronval formally denies the existence of the acarus scabiei, of the "sarcopte" that succeeds it, and of all psoric animal-

* Recherches et Observations sur la Gale, faites a l'Hôpital Saint Louis, à la clinique de M. Lugol, pendant les années 1819, 1820, et 1821. A Paris, 1821, 8vo.

cules* whatever. Dr. R. is likewise quite satisfied of M. Lugol's having definitively proved, by numerous and well conducted experiments, that the itch may be cured more or less speedily by stimulants applied to the skin, under the form of baths, fumigations, lotions, and frictions (very good Dr. R. but what are the ingredients ?); that it will even yield to baths of aqueous vapour; and, that the internal treatment to which so much importance is attached by some practitioners, is absolutely useless, except in cases where the long duration, the extent, and the intensity of the disease, become a consideration why it ought not to be imprudently suppressed.

Dr. Biett has the charge of patients afflicted with tetters, and has made a multitude of interesting experiments on the different substances employed for the cure of these eruptions: he is also engaged in a course of curious and important researches relatively to epilepsy and some other affections whose obstinacy has often "reduced physicians to despair."

*Hôpital des Vénériens.*—M. Cullerier and his nephew, with Professor Bertin, are physicians to this institution, in which syphilis and the venereal affections are exclusively treated. Every form and variety of such diseases may be observed in these wards, and they afford moreover the best opportunities of ascertaining the effects of the various means employed for their cure.

Notwithstanding the different forms, says Dr. B. which the venereal disease assumes, the principles of its treatment are and ought always to be the same: we modify them only, he adds according as the affection is primary or consecutive: the local treatment varies according to the symptoms and the different degrees of their intensity. In syphilis, mercury is considered as a specific; and the cases, we are told, wherein it has not succeeded, form exceptions which ought not to weaken the general rule. Experiments made in Spain and Italy, howeve., have shown that vegetables, especially such as are sudorific, and a warm climate, have removed the disease without the aid of mercury. In combating the primary symptoms, Van Swieten's liquor in doses containing half a grain of the muriate, every day, with barley water, or other diluent drink, constitutes the common treatment in this hospital. When

* This opinion, drawn from observation, is so much at variance with that advanced, from observation also, by M. Galés, that the question must be considered as one of great interest both with regard to medicine and natural history. At no distant period, we propose bringing it under the consideration of our readers.

patients have obstructions in the chest, or a predisposition to pulmonary consumption; when the abdomen is the seat of some irritation; and, when the liquor determines pain in the stomach with vomiting, M. Cullerier has recourse to mercurial pills or inunction. To persons having secondary symptoms, M. C. exhibits the liquor with success so remarkable, that if the disease resists it and continues making progress, we may conclude there is negligence on the part of the patient in applying the treatment. Frictions are held by M. Cullerier to be an energetic remedy for constitutional syphilis, and he employs them indifferently with Van Swieten's liquor. In inveterate cases, he administers along with this, a sudorific ptisan simply, or sweetened with a diaphoretic syrup: to this he joins bark, or an anti-scorbutic syrup, for persons of a debilitated constitution.

M. Cullerier exhibits the "Tisane de Feltz" (afterwards described) with a degree of success truly surprising; and, on this subject, has accumulated many extremely interesting facts. He prescribes this ptisan when the consecutive symptoms, such as exostoses, osteocopic pains, periostoses, serpiginous pustules, ulcerations of the soft parts, caries of bones and of the cartilages of the nose and mouth, after having yielded several times to the anti-venereal liquor or frictions, continue to reappear. This practice, we are informed, almost invariably produces a cure, even in cases of considerable standing, or at least, when the disease has not occasioned very great morbid alterations. In the hands of this physician, the hydro-chlorates (muriates) of gold and platina have not answered the pompous announcements of their inventors as anti-syphilitic remedies: he has therefore, discontinued their use. If our recollection does not mislead us, M. C's. own opinion regarding the effects of these medicines, must have undergone something like a change. He also esteems the bath, holding a solution of the deuto-chloride of mercury, as a most energetic application for the removal of venereal symptoms: but he does not prescribe it in the hospital, because the mode of administering it requires the greatest precaution. His management of local symptoms is simple, and has little particular in it: it consists chiefly of cleanliness, rest, mild or mercurial washes and ointments, with the potential, and sometimes the actual cautery: his principal reliance is on the influences of a constitutional treatment.

*Maison de Santé.*—Professor Dubois is the chief medical officer of this establishment. Although principally engaged in the practice of surgery, he is consulted by a great number of patients for the relief of internal diseases. He entertains peculiar opinions on certain points of practice, and conceives them to be established by the facts of long experience. In scrofulous, scorbutic, and similar affections, he employs a tonic treatment, and considers salivation produced by mercurial friction as the surest means of subduing inveterate syphilitic symptoms. His opinions, in general, are nearly Brunonian: he frequently uses revulsives, tonics, and stimulants: bleeding is practised by him with an extreme reserve, even in cases where most physicians regard it as indispensable: he rarely prescribes general bleeding in acute inflammation, whether of membranous or parenchymatous organs: on such occasions, he confines his interference, to the application of leeches, followed by blisters.

*Hospice de la Maternité.*—Professor Chaussier, is physician to this fine establishment, to which access, at least an habitual one, is withheld from students and foreign practitioners. An investigation of the nature of puerperal affections almost constantly engages M. C's attention. Reprobating all systems, he founds the treatment of disease, in his profound physiological knowledge: he uses a very small number of medicines, and prefers those whose properties have been ascertained: his trials of new substances are always conducted with philosophical reserve.

*Hôpital Saint-Antoine.*—This is one of the most healthy institutions in the capital: it receives persons suffering from acute diseases and those chronic affections that have not a hospital appropriated for their reception. M. Beauchéne is the chief surgeon in this house, and doctors Kapeler and Lullier Winslow the physicians: the former is much engaged with therapeutical researches; and, M. Ratier has been told, employs with advantage the contra-stimulant practice of the Italian school. This method is adopted by a considerable number of practitioners, and there are several hospitals where, in the course of a few hours, 12, 20, 30, and even 40 grains of emetic tartar, are exhibited to one patient, and in a quantity of fluid infinitely small in proportion to the dose of antimony. It is very singular, but not the less true, says M. R. that such patients experience no inconvenience from this treatment; neither do they vomit or manifest any remarkable primary symptoms. The doctor submits these facts to physiological readers, and abstains from expressing his own sentiments.

*Hôpital Necker.*—This small establishment, situated at a distance from the centre of the metropolis, is appropriated to the reception of chronic diseases; but was little frequented till M. Laennec, its physician, drew attention to himself and this hospital by his admirable investigation of thoracic diseases, and the new method of examining the state of the chest which he introduced into practice. Dr. L. cultivates with great assiduity the study of pathological anatomy, and makes occasional experiments with contra-stimulant medicine.

*Hôpital de la Salpêtrière.*—Into this large establishment are admitted females advanced in years, or affected with incurable diseases and infirmities of any kind: women suffering from mental alienation are likewise received into it; but they have separate appartments. The latter part of the institution is under the care of Professor Pinel and Doctor Esquirol, who now, in a great measure, supplies the place of his venerable master: the surgical wards are entrusted to Professor Lallemant, and the infirmary for sick and infirm females to M. M. Rostan and Ferras. Students generally attend the Salpêtrière with the object of investigating the nature of mental alienation, on the varieties of which Dr. Esquirol gives a course of clinical lectures.

This eminent physician employs two sorts of remedies for the relief or cure of intellectual aberration. The one consists of means intended to modify the organ of mind *(the brain)* by the exercise alone of its functions: these he calls *intellectual and moral* agents, the others are *physical*, and are taken from the ordinary resources of the materia medica, and have for their object the fulfilment of different curative indications.

*Moral and Intellectual Treatment.*—This combines five indications. 1st. To remove the insane from the objects which have excited their disease, and from parents or servants they dislike, or whom they are unwilling to obey. 2nd. To treat them with gentleness; but, at the same time, with necessary firmness. 3d. To arrange them, in the institution, in a manner so as to prevent them from injuring themselves, and also to contribute to their cure: to place the furious at a distance from the other patients: to shut up very agitated persons in obscure or even perfectly dark chambers; and, not to compel those who experience an immoderate heat to cover themselves with too warm or too light garments. 4th. To separate entirely the convalescents from all others, by causing them to pass successively into cells more tranquil in proportion to the advances of their cure: to withdraw from these cells, such persons as seem disposed to have a new accession or exacerbation of their malady: and to encourage those, whose reason is not altogether disordered or overthrown, to engage in walking, working, or amusement. 5th. Never to employ, but as means of restraint or punishment only, the strait-waistcoat, confinement in a cell, removal from one division of the house to another, or pumping of cold water; never to have recourse to blows, chains, or harsh measures of any kind: to superintend the first interviews of the lunatics with their relatives and friends: occasionally, to excite intense mental emotions, by a surprise, an alarm, or an unmerited reprimand: to replace one passion by another, religion for love: never to compromise professional dignity by entering into idle discussion with the insane: to guard particularly against exciting them to anger or rage, by directly contradicting their ideas and their passions, or in any other way: to promote social intercourse of the convalescent patients: to exercise the most active vigilance over such as have a tendency to suicide, even for a long time after they *appear* to have recovered from this fatal propensity; otherwise the physician will incur the risk of being made the dupe of dissimulation at once profound and concealed with extraordinary prudence: to watch and isolate patients addicted to masturbation or to "*un vice plus honteux*:" to avoid recalling to the recollection of those who are recovering, the extravagancies of their insanity or any circumstances that give them uneasiness, especially when they themselves are not the first to make such things the subject of conversation: and, in fine, to institute every precaution against the recurrence of causes tending to induce a relapse.

*Physical Treatment.*—This comprehends the use of hygienic and medicinal applications.

*Hygienic Means.*—These are,—protecting the insane from the influences of excess in the temperature, and of sudden variations of the atmosphere, by keeping them in apartments properly heated during the winter, and preventing them from going bare-footed into icy water or the snow, and particularly by defending them, in the heat of summer, from the action of the solar rays: washing every day and airing the cells of such as are negligent of cleanliness: securing in their beds, during the night, those who have a mania for lying on the ground, and paralytic patients that throw themselves involuntarily out of bed; or preferably, putting the latter into cribs where they can be kept without restraint:—providing carefully that all of them be covered in bed, especially in cold weather, so as to prevent their being frost-bitten in the extremities; nothing is better for this purpose than placing a straw mattrass above the coverlet:—giving, in all cases, one or two baths daily, for the sake of purifying their persons:—cutting off the hair, on all occasions where there is much heat in the head and an habitual state of irritation, when there is congestion in that part, and when, the patient being furious, it is impossible to keep the hair clean:—distributing abundance of wholesome food, four times in the day; and above all, seldom refusing drink or food to the patients, even though they desire it in the night:—exhibiting nutritive clysters to those who obstinately refuse aliment; or, when unavoidable, injecting milk, soup, and sometimes wine into the stomach by means of a proper instrument.

*Curative Indications.*—Insanity is commonly of long duration: its nature in many instances, at least, is difficult to be understood: the indications of treatment founded on this knowledge are, therefore, almost always difficult to be apprehended, often little or not at all determinate: in the Salpêtrière, scarcely one third of the patients, who present no symptoms of being incurable, are recovered. For these reasons, a physician ought, at all times, to be very circumspect: he should rather refrain from prescribing remedies than run the risk of exhibiting them without indications, and thereby producing effects quite different from those he would desire: he ought especially to guard against being imposed upon by the violence of certain symptoms, and against believing that the cause of the disease has relation to their intensity. In two circumstances only, the physician should act with vigour; at the commencement of the seizure, and when the use of means rationally indicated has been continued so long as to make the incurableness of the malady to be dreaded. When loss of reason has become confirmed; when, after several years duration, it has degenerated into absolute insanity; and, especially, when it is complicated with paralytic debilities, then it will be in vain to persevere in attempting a cure: then, moreover, the physician's chief duty will consist in trying to prolong existence by preventing or removing morbid states of the brain, or other accidents by which the patient's life may be endangered. Let us now briefly consider some of the principal remedies employed by Dr. Esquirol in the treatment of insane persons.

*Sanguineous Evacuations.*—Bleeding has often been abused, in consequence of the physician's confounding a general plethora and congestion of the brain, with the state of frenzy, and his being thus led to believe the possibility of subduing the frenzy by diminishing the mass of blood. In this, however, there is much deception. Plethora and cerebral congestion have characters quite distinct from those of frenzy or furious insanity: they often exist independently of the latter, and it is altogether vain to expect being able to subdue permanently a furious lunatic by bleeding him to the utmost: the insane often become more frantic after losing blood, and they only cease to be so when forcibly restrained. Paroxysms of maniacal frenzy run the same course, with or without sanguineous depletion: but when there exists a genuine plethora or a state of cerebral congestion, especially at the beginning of the disease, there should be no hesitation in abstracting blood, by cupping, leeching, or a general bleeding. When suppression of an habitual sanguineous discharge has taken place, the two former remedies are to be applied near the parts which were the seat of such periodical evacuation.

*Baths.*—Tepid bathing; the cold seldom is employed in the Salpêtrière: it has many advantages in tranquillizing symptoms in the insane.

*Douches.*—This term means purifying or dashing cold water over the body. The practice is rarely adopted, and never without the greatest circumspection, particularly when there is violent head-ache, or excessive heat in the head. It is oftenest used for overcoming patients who have taken some fatal resolution, as for instance to destroy themselves by hunger. Cold affusions on the head are very tranquillizing and may be made without danger. In cerebral congestions, bleeding from the jugular vein or leeching of the neck is preceded by a tepid bath wherein the patient is immersed for a determinate period, during which refrigerant applications are placed on the head.

*Emetics.*—In certain cases of oppression and insensibility with dejection and irritability, vomiting has beneficial effects by the shock it communicates to the general system; but it ought to be produced with the greatest caution, on account of the determination of blood to the head which almost always prevails in insanity.

*Purgatives.*—They are generally indispensable, both as the means of overcoming constipation, which is a symptom of the highest importance and should never be neglected, and of establishing a salutary derivation (of the humours, we suppose) to the intestinal canal.

*Derivatives.*—These, directed towards the bowels and the skin, are in frequent use, and very advantageous in the greater part of mental alienations, especially such as succeed parturition: in these cases, purgative lavements and diaphoretics are preferable.

*Moxas, Blisters, Setons.*—Several cures have been effected by the application of moxa to the head: in one instance, it excited fatal inflammation of the brain: often after its use, the disease remains unchanged. Nearly similar results are obtained from applying the actual cautery. Setons in the neck, and blisters have been employed on the same principles and with the same precautions.

*Narcotics.*—Such remedies are rarely useful at the commencement of insanity: often, they induce no effect whatever; and, when they do procure sleep, they leave the delirium unabated.

*General Means.*—Acidulous drinks are given liberally to the patients: mucilaginous and nitrated ones, during the period of irritation. Mild tonics are exhibited when it is found requisite to support the vital forces: medicine, in fine, is administered according to the general rules of therapeutical science.

Here we must stop for the present. In our next number we shall conclude the picture of the Parisian Hospital practice.

